# Domestic pigs (*Sus scrofa*) engage in non-random post-conflict affiliation with third parties: cognitive and functional implications

**DOI:** 10.1007/s10071-022-01688-4

**Published:** 2022-11-08

**Authors:** Giada Cordoni, Marta Comin, Edoardo Collarini, Carlo Robino, Elena Chierto, Ivan Norscia

**Affiliations:** 1 Department of Life Sciences and Systems Biology, University of Torino, Turin, Italy; 2 Department of Public Health Sciences and Pediatrics, University of Torino, Turin, Italy

**Keywords:** Reconciliation, Triadic contacts, Anxiety-reduction, Social appraisal, Intrinsic-extrinsic emotional regulation

## Abstract

In social mammals, conflict resolution involves the reunion of former opponents (aggressor and victim) after an aggressive event (reconciliation) or post-conflict triadic contacts with a third party, started by either opponent (solicited-TSC) or spontaneously offered by the third party (unsolicited-TUC). These post-conflict strategies can serve different functions, including consolation (specifically when TUCs reduce the victim’s anxiety). We investigated the possible presence and modulating factors of such strategies on semi-free ranging pigs (*Sus scrofa*; *N* = 104), housed at the ethical farm *Parva Domus* (Cavagnolo, Italy). Kinship was known. Reconciliation was present and mainly occurred between weakly related pigs to possibly improve tolerant cohabitation. Triadic contacts (all present except aggressor TSCs) mostly occurred between close kin. TSCs enacted by victims reduced neither their post-conflict anxiety behaviors nor further attacks by the previous aggressor, possibly because TSCs remained largely unreciprocated. TUCs towards aggressors did not reduce aggressor post-conflict anxiety but limited aggression redirection towards third parties. TUCs towards the victim reduced the victim but not the third-party’s anxiety. However, TUCs may also provide inclusive fitness benefits to third parties by benefiting close kin. In sum, pigs engaged in non-random solicited/unsolicited triadic contacts, which suggests that pigs might possess socio-emotional regulation abilities to change their own or others’ experience and elements of social appraisal, necessary to detect the emotional arousal of relevant others and (in case of TUCs) take the agency to restore homeostasis.

**Supplementary Information:**

The online version contains supplementary material available at 10.1007/s10071-022-01688-4.

## Introduction

The *Social Intelligence Hypothesis* posits that social cognition in group-living vertebrates develops in response to the structural complexity of the group and, consequently, to the challenges that the individuals face for social living (Byrne and Whiten 1988; Dunbar and Shultz 2007). According to the relational model proposed by de Waal (2000) one of the main challenges of social animals is to manage the conflicts over resources that inevitably arise within groups and that—if not resolved—may lead to group disruption; thus, an aggressive event can have cascading consequences that affect all group members (Schino and Sciarretta 2015; Pallante et al. 2016).

**Fig. 1:**
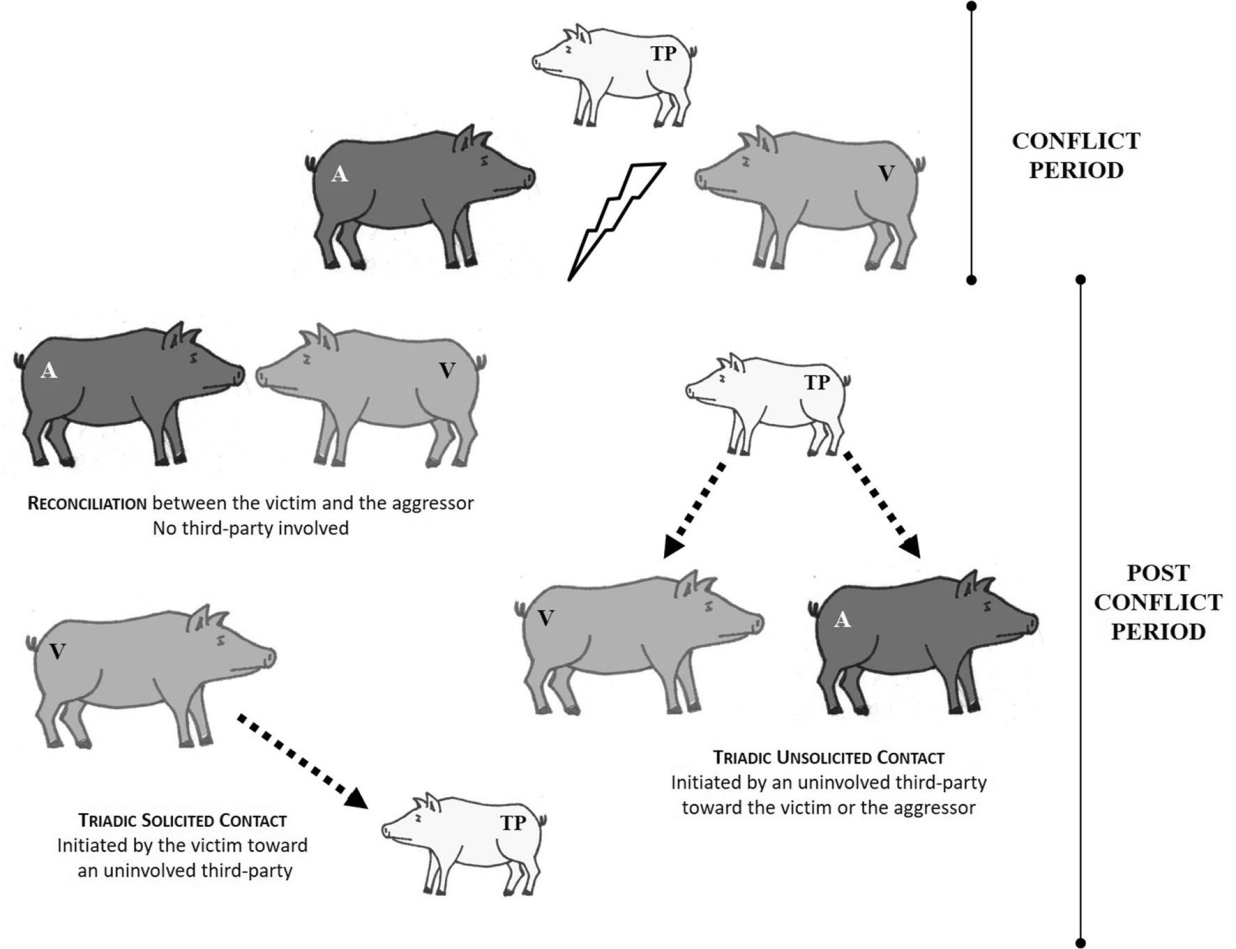
Graphical summary of post-conflict mechanisms observed in the study pig. Legend: *A* Aggressor, *V* Victim; *TP* Third-Party

Several species have developed post-conflict behavioral strategies to preserve group integrity (see Fig. 1), namely: (i) *reconciliation*, which is defined as the first affiliative contact exchanged between the two opponents right after the end of the aggression (de Waal and van Roosmaleen 1979); and (ii) *triadic affiliation*, which is defined as the first affiliative contact exchanged between one (or both) of the opponents and an uninvolved third party (Romero et al. 2009, 2011). In particular, triadic affiliations can be divided into two types: (i) ‘*solicited’* if initiated by the victim or the aggressor and directed towards a third party (de Waal and Aureli 1996; de Waal 2000; Palagi and Cordoni 2009); and (ii) ‘*unsolicited’* if spontaneously initiated by a third party and directed towards the victim or the aggressor (de Waal and Preston 2017). These two types of triadic affiliation can underlie different functions and cognitive abilities (Fraser et al. 2009; Romero et al. 2009, 2011; Cordoni and Palagi 2015; de Waal and Preston 2017).

Here below we define and detail the above-mentioned post-conflict strategies. Because no direct linkage between psychological processes and behavioral manifestations can be unambiguously delineated (Leavens et al. 2019), we reported the main hypotheses on the cognitive mechanisms that might be (although not exclusively) implicated in post-conflict behavioral strategies.

### Reconciliation

Reconciliation is technically defined as the first affiliative contact exchanged between the former opponents (i.e. aggressor and aggression's recipient or victim) within a few minutes (generally 2-min) after a conflict (de Waal and van Roosmaleen 1979; Cordoni et al. 2006; Cordoni and Palagi 2008; Cordoni and Norscia 2014). This phenomenon has been described in various social species, including birds (e.g. parrots, Ikkatai et al. 2016; craws, Sima et al. 2018), a marsupial species (red-necked wallabies, Cordoni and Norscia 2014) and different placental mammals, particularly human and non-human primates (for review see Norscia and Palagi 2016). Reconciliation is a dyadic interaction between two social agents (the former opponents) that might require elements of second-person (or hereafter, ‘second-subject’) participatory capabilities (in the broader sense; *sensu* de Jaegher et al. 2010).

From a functional point of view—among other effects (Aureli 1997; Romero et al. 2009; McFarland and Majolo 2011)—reconciliation can work in repairing the social relationship between the opponents potentially damaged by the aggression, especially with valuable partners such as kin or friends (*Valuable Relationship Hypothesis*, de Waal and Aureli 1997; Wittig and Boesch 2005).

From a cognitive point of view, it has been hypothesized that reconciliation might be based on individual recognition and implicit memory of previously encountered subjects (Cords and Thurnheer 1993; Aureli et al. 2002). These abilities might allow animals to behave appropriately and establish or maintain preferential social bonds with specific companions (Gheusi et al. 1994; Massen 2017; Yorzinski 2017). Hence, reconciliation may be not randomly distributed among dyads of opponents and be skewed by the social bond between aggressor and victim (de Waal and Aureli 1997). This might require the further ability to attribute different social values to others (Swallow and Kuiper 1988; Taylor et al. 1995).

### Triadic affiliation

*Triadic solicited contacts* occur when one of the former opponents (victim or aggressor) approaches an uninvolved third party and starts an affiliative contact with her/him (de Waal and Aureli 1996; de Waal 2000; Palagi and Cordoni 2009). From a functional point of view, solicited contacts can indeed regulate the former opponent’s experience because they can (i) decrease the probability to receive further aggression from other group members (*Victim Protection Hypothesis*, Palagi and Norscia 2013; Palagi et al. 2014) and/or (ii) reduce self-anxiety (especially if victims; *Self Anxiety Reduction Hypothesis*; Palagi and Cordoni 2009; McFarland and Majolo 2012; Puga-Gonzalez et al. 2014).

From a social cognition point of view, it can be hypothesized that solicited contacts—although involving a third party—may be still considered dyadic relations (*sensu* Freiwald 2020). To occur, solicited contacts may not necessarily require that a non-participant subject witnesses the interaction between two other social agents (conflict opponents). In this respect, solicited contacts might be a form of *intrinsic* social regulation (*sensu* Zaki and Williams 2013), as an individual (the former opponent) initiates a social contact to possibly regulate *its own* experience. Because the response of the contacted subject can make a difference in such regulation (Zaki and Williams 2013), the contacted third party may not be a random subject (but for example a socially close one; Cordoni et al. 2006; Palagi et al. 2008).

*Triadic unsolicited contacts* occur when an uninvolved third party spontaneously approaches and starts an affiliative contact with one of the former opponents (de Waal and Preston 2017).

The functional effect produced by unsolicited triadic contacts can depend on which one of the former opponents (aggressor or victim) is approached (Romero et al. 2009, 2011; Cordoni and Palagi 2015; Pérez-Manrique and Gomila 2018). When unsolicited triadic contacts are offered to the former aggressor they mostly reduce the risk of further attacks (*Appeasement Hypothesis*; Das 2000; Romero et al. 2011; Cordoni and Palagi 2015). When triadic contacts are engaged with the victim, they may protect the victim against renewed aggression (*Victim Protection Hypothesis*) and reduce its anxiety (*Consolation Hypothesis*; de Waal and van Roosmaleen 1979; Fraser et al. 2008; Fraser and Bugnyar 2010; Romero and de Waal 2010; Palagi and Norscia 2013). Specifically, unsolicited triadic affiliation directed to the victim has defined ‘consolation’ if and only if it induces a decrease in anxiety levels in the victim in the few minutes following the affiliative interaction (Fraser et al., 2009; de Waal and Preston 2017).

From a social cognition point of view, it may be hypothesized that unsolicited contact might imply a further cognitive step because the interaction between two social agents (the opponents while fighting) is detected and processed by a non-participant subject (uninvolved third party). Recent neuroscientific investigation has found that in primates, for example, direct social interactions and observed social interactions are processed—at least in part—by different brain cortical areas (Freiwald 2020). Furthermore, different regulatory mechanisms may be present (*as per* Zaki and Williams 2013) because it is the bystander that takes agency and actively approaches either one of the former opponents, to possibly change its own experience (intrinsic regulation) and/or the experience of the contacted subject (extrinsic regulation).

When the spontaneous triadic contact by third-party works in reducing the emotional arousal in the contacted individual further cognitive mechanisms may be hypothesized, including possible intrinsic motivation and prosocial function (de Waal and Preston 2017). The process can require elements of social appraisal, through which an individual’s appreciation of a social partner’s emotional behavior toward a shared referent regulates the individual’s subsequent behavior in relation to such referent (Walle et al. 2017), in this case the aggressive event. Perceiving others’ arousal can indeed generate arousal in the observer and induce other-oriented behavior via emotional resonance (Decety et al. 2016). Both the Mirror Neuron System and the Perception-Action Mechanism (PAM) foresee that shared representations of actions may lead to shared representations of the emotions underlying such actions, with the process being modulated by the action goal (MNS: Schütz-Bosbach and Prinz 2015; Caruana 2019) and the observer’s experience (PAM: de Waal and Preston 2017). As a first consequence, the resulting behavior of the observer can be implicitly aimed at reducing the divergence between the observer’s actual internal state and the observer’s prediction of the other subject’s emotional state (Prochazkova and Kret 2017). A further consequence is that unsolicited post-conflict contacts may be influenced by individual experience and occur more frequently between closely bonded subjects compared to other dyads (Palagi et al. 2020).

### Focus of the study and predictions

In this study, we provide a comprehensive investigation of the post-conflict mechanisms possibly present in semi-free ranging domestic pigs (*Sus scrofa*), a cognitively advanced species that is able to discriminate familiar individuals and objects, shows sensitivity to the internal states of others and proactively responds to others’ distress (Reimert et al. 2013, 2015; Marino and Colvin 2015; Goumon and Špinka 2016; Camerlink et al. 2018; Norscia et al. 2021a, 2021b). Domestic pig reared under semi-natural conditions can perform social behavioral patterns that are typical of its wild counterpart (i.e. wild boar; Jensen, 1986; Stolba and Wood-Gush, 1989). A large array of social interactions between pigs rely on olfaction: nose-to-body and nose-to-nose contacts serve a social exploration and recognition function in affiliative contexts (Camerlink and Turner, 2013; Camerlink et al. 2014). Vocal and body postures are used for inter-individual interactions and communication as well (d’Eath and Turner 2009; Horback 2014). In extensive farms, sows—usually kin related—can form small sub-groups (sounders) including their offspring. Adult females can synchronize their foraging and resting activities and can cooperate in piglet defense (Stolba and Wood-Gush 1989; d’Eath & Turner 2009). Sows leave only temporarily their sounder at the end of pregnancy (about 115 days) to search for a suitable farrowing place and build the nest (Jensen 1986). Mature males can stay in proximity of their conspecifics especially during the mating period but young adults can form stable bachelor groups (Jensen 1982, 2002; Stolba & Wood-Gush 1989; d’Eath & Turner 2009; Dalmau et al. 2020). The different ranking positions are quickly defined through aggression that is generally won by heavier individuals (D'Eath 2002; Andersen et al. 2004; Norring et al. 2019). In well-established groups, subordinates try to limit aggression by avoiding dominant individuals (Jensen 1982). Nevertheless, aggressive behavior can occur and cause an increase in anxiety (Norscia et al. 2021a, b), physiological stress (Arey and Edwards 1998) and social uncertainty (Cords and Aureli 2000) in group members.

Here, we resume the functionalist framework common to animal post-conflict studies, but we also provide possible insights on whether the presence and modulation of different post-conflict behaviors might support the proposed hypotheses on the cognitive abilities possibly underlying such behaviors. Hence, we tested some hypotheses on post-conflict management in this species and we formulated the following predictions.

### Prediction 1—Reconciliation

Pigs are able to distinguish familiar from unfamiliar subjects in large groups and they can also recognize group fellows after several weeks of separation (Kristensen et al. 2001; Turner et al. 2001; McLeman et al. 2005). Thus, domestic pigs may be capable of individual recognition and implicit memory to engage with specific group mates in post-conflict reunions. If so, reconciliation should occur in *Sus scrofa* (*Prediction 1a*). In semi-free ranging and feral pigs, the main social unit is usually formed by kin-related individuals (i.e. generally, two or four related sows with their most recent litters and juvenile subjects of previous litters) that establish preferential social bonds (Jensen 1982, 2002; Graves 1984; Stolba and Wood-Gush 1984; D’Eath and Turner 2009). Hence, pigs may be able to attribute different social values to others (Goumon et al. 2020). If so, the reconciliation should not be randomly distributed across dyads of opponents. In this view, if reconciliation is more frequent when the conflict occurs between closely related than unrelated or less closely related pigs, we can support the *Valuable Relationship Hypothesis* (*Prediction 1b*).

### Prediction 2—Triadic solicited contacts

The presence or proximity of other conspecifics has an effect on how pigs cope with stressful situations (Reimert et al. 2014). Thus, pigs might have elements of implicit regulation (sensu Zaki and Williams 2013). If so, we predict to find solicited triadic contacts in pigs (*Prediction 2a*). Since (i) the effectiveness of social regulation also depends on which subject is contacted (Zaki and Williams 2013) and (ii) pigs can establish preferential social bonds with certain fellows (Jensen 1982, 2002; Graves 1984; Stolba and Wood-Gush 1984; D'Eath and Turner 2009), we predicted that solicited triadic contacts occur most frequently between closely-related pigs (*Prediction 2b*). In semi-free-ranging pigs post-conflict affiliation also involves bystanders and social contacts can reduce individual anxiety levels measured via self-directed behaviors (Norscia et al. 2021b). Based on these evidences, if solicited affiliation reduces the anxiety in the opponents, we can support the *Self Anxiety Reduction Hypothesis* (*Prediction 2c*). Moreover, if solicited contacts—via this calming effect—reduce the levels of renewed aggression in the victim, we can support the *Victim Protection Hypothesis* (*Prediction 2d*).

### Prediction 3—Triadic unsolicited contacts

Pigs show abilities of individual and object discrimination and understanding of others’ cues (Marino and Colvin 2015; Camerlink et al. 2018). Moreover, pigs are sensitive to the physiological and emotional state of others (Norscia et al. 2021a), can respond to others’ distress via making contact or moving in proximity (Reimert et al. 2013; Goumon and Špinka 2016; Norscia et al. 2021b) and show emotional contagion especially in negative situations (Reimert et al. 2015). Hence, pigs might have regulatory mechanisms and elements of social appraisal (*sensu* Zaki and Williams 2013). Moreover, in pigs post-conflict arousal experienced by both former opponents and bystanders is buffered by affiliation, which restores baseline levels within three minutes (Norscia et al. 2021b). Hence, we expected that pigs would show unsolicited triadic contacts (*Prediction 3a*). Moreover, we expected that pigs would especially engage in unsolicited triadic contact with closely-related individuals to make social regulation more effective (*Prediction 3b*).

Finally, if unsolicited triadic contacts directed to the aggressor reduce the probability of renewed attacks on other group members or on the victim, we can support the *Appeasement Hypothesis* (*Prediction 3c*) and the *Victim Protection Hypothesis* (*Prediction 3d*), respectively. Moreover, if unsolicited affiliation reduces the anxiety levels in the victim, we can support the *Consolation Hypothesis* (*Prediction 3e*).

## Methods

### The study group

The study was carried out on a group of semi-free ranging domestic pigs composed of 104 adult individuals (54 males and 50 females, 7–22 months of age) belonging to three different breeds: Parma Black, Large White and Piedmont Black. The pigs were housed at the ethical farm “*Parva Domus*” (Cavagnolo, Turin—Italy) in a woodland natural area of about 13 ha. Ethical farms make important efforts to enhance animal welfare by rearing animals in a natural or semi-natural environment where they are able to (i) freely move and behave according to their specific behavioral repertoire, (ii) integrate their diet with the natural food and (iii) follow their natural day/night cycle. The pigs under study received food pellets (Ciclo Unico P, SILDAMIN^®^) each morning between 8:30–10:30 am but they also freely foraged throughout the area. The water was available* ad libitum*. The males living in the group were castrated during their first three days of life, while the reproductive male was separated from the rest of the group. Due to the summer culling suspension (June/September) and the subsequent low culling rates (usually one individual per week), all but eight pigs were available for the whole data collection period.

### Kinship determination and genetical analyses

Owing to controlled reproduction, kinship (when present) varied from second cousins to full siblings. The different breeds, sizes, and marks allowed the reliable identification of the different generations. However, different mothers and fathers could be related (e.g. siblings or cousins). Therefore, to distinguish between distantly related animals from more closely related ones, genetic analyses were carried out on 31 pigs (2–3 individuals sampled from different sibling generations) at the forensic genetic lab of the Department of Public Health Sciences and Pediatrics (University of Torino). Pig’s hair samples were collected by the farmer during the usual weekly visit of a veterinarian. Hair samples were grasped using small tweezers (similar to used in human eyebrows). DNA was extracted by hair bulbs (collected during the study period) via QIAmp DNA Investigator Kit (Qiagen; www.qiage n.com) following the provider’s protocol. 11 autosomic STRs were amplified via multiplex PCR Animal Type Pig PCR amplification kit (http://www.bioty pe.de; Biotype AG, Dresden, Germany). Genetic profile typing was obtained via capillary electrophoresis with SeqStudio system (Thermo Fisher Scientific; www.thermofisher.com). Allele frequencies and kinship index (0.08) were set on the basis of a mixed sample of domestic pigs (*n* = 412), consisting of commercial lines commonly used in the production process (Caratti et al. 2010). The mutation rate for all markers was set at 0.002. For each possible dyad of pigs an unspecific kinship search was performed using Familias 3.1.5 “Blind Search” Module (Kling et al. 2014). Likelihood ratio (LR) was calculated for sibling, half-sibling, 1st cousin, and 2nd cousin relationships, scaled versus unrelated. Relationship was assigned according to the maximum LR value observed among the tested relationships.

### Data collection

We collected video data from June to November 2018 on a daily basis spanning morning and afternoon (7:00 am until 5:00 pm). The videos were recorded by two operators (E.C., M.C.) and a field assistant via Panasonic HC-V380/V180 and Sony HDR-PJ240E cameras. In total, 224 videos were collected corresponding to 43.0 hours of video observation (mean hour/subject 4.84 ± 1.85 SD). During the video recording, we maintained a wide zoom to improve the data collection (e.g. recording more conflicts that occurred concomitantly). The videos were then analyzed, frame-by-frame when necessary, via freeware VLC 3.0.6 and extension Jump-to-Time. Before starting the systematic video analysis I.N. and G.C. supervised M.C. and E.C. in a training period of 24 h to reach an interobserver reliability score (Cohen’s k) of at least 0.81 for aggression, post-conflict affiliation, and anxiety-related behaviors (strong agreement *sensu* McHugh 2012; for the definition of behavioral items see Table S1). The Cohen’s k value was measured using the R function “cohen.cappa” and libraries “irr” and “psych” (R version 3.5.3). From the video analysis, we extracted 216 aggressive events including 104 pigs. However, not all pigs engaged in conflicts or were present in post-conflict contexts (plus in different cases the victim or aggressor or both the opponents could move out of sight) or in all situations (reconciliation, TUC and TSC). Moreover, eight out of 104 pigs were culled during the study period (see *The study group*) and for these animals we did not have data in all contexts/situations. For all these reasons, the sample sizes of the different analyses are different.

### Operational definitions

For each conflict, we recorded the identity and features (e.g. gender, age, kinship) of the aggressor (i.e. the initiator of the conflict), victim (i.e. the aggression’s recipient) and third-party or bystander (i.e. a pig not involved in the conflict that at the end of the aggression engaged in an affiliative contact with one of the former opponents). For data analyses, dyads were classified as weakly related (1st or 2nd cousins and unrelated individuals) and closely related (half- and full-siblings). The triadic affiliation was distinguished in solicited (TSC, Triadic Solicited Contact) if started by the victim/aggressor and unsolicited (TUC, Triadic Unsolicited Contact) if started by the third party (Fraser and Aureli 2008; Fraser et al. 2009).

To evaluate the occurrence of reconciliation (*Prediction 1a*), TSC (*Prediction 2a*) and TUC (*Prediction 3a*), with either victim or aggressor we employed the standard PC-MC method used in post-conflict studies on animals (de Waal and Yoshihara 1983; Arnold and Aureli 2010). After each agonistic event, we followed the opponents for a 3-min Post-Conflict period (PC). We used a 3-min time window as it has been previously demonstrated in the same study group that anxiety-related behaviors dropped within such time window (Norscia et al. 2021b). For each PC, a corresponding 3-min Matched Control observation (MC) of the behavior of the same individuals is recorded. This observation is usually carried out on the next possible day at the same time, and in the same social (presence of at least four individuals other than the opponents within max 20 m) and environmental context (same weather and time ± 1 h) on the original victim and aggressor, in absence of conflict in the previous 10 min. For both PC and MC, we recorded (i) the time gap (measured as mm:ss,00) between the starting of the PC or MC and the occurrence of the first affiliative contact (if present) between victim-aggressor, bystander-victim and bystander-aggressor, (ii) the type of first affiliative contact (the post-conflict affiliative contacts recorded were instantaneous events *sensu* Altmann 1974, such as nose-nose or nose-body contacts; see Table S1), and (iii) the initiator of affiliation. By this procedure, we obtained an equal number of PC and MC observation pairs that were compared with respect to the length of the time gap between the starting of PC/MC and the first affiliative contact between victim-aggressor, bystander-victim and bystander-aggressor. Pairs were classified as attracted—when the time-gap was shorter in PC than MC or affiliative contact was present only in PC-, dispersed—when the time-gap was shorter in MC than PC or affiliative contact was present only in MC-, and neutral—when the time-gap was equal in both PC and MC or affiliative contact did not occur in both PC and MC. In all our analyses we included pigs with at least 3 PC-MC pairs so that they could have at least one pair per type (attracted, dispersed and neutral pairs; Schino et al. 1998). As per de Waal and Yoshihara (1983), the presence of reconciliation and/or triadic affiliation can be confirmed if the number of attracted pairs is significantly higher than the number of dispersed pairs at the individual level. We evaluated individual conciliatory levels by measuring the Corrected Conciliatory Tendency defined as ‘attracted minus dispersed pairs divided by the total number of PC-MC pairs’ (Veenema et al. 1994). Individual Corrected Conciliatory Tendencies were used to determine the mean group Corrected Conciliatory Tendency. We used the same formula to calculate the Triadic Contact Tendency for either solicited and unsolicited affiliation.

A previous report indicates that in the same study population the level of specific self-directed behaviors (i.e. head/body shaking, vacuum-chewing, yawning, and scratching/body rubbing; Table S1) can be used as reliable anxiety indicators (Norscia et al. 2021b). Using such indicators, we investigated if, in absence of previous reconciliation, triadic affiliation could reduce anxiety levels in the study subjects. In particular, we compared the levels of anxiety-related behaviors of aggressor, victim and (for TUCs toward the victim) third parties, in the following three post-conflict conditions: (1) no triadic affiliation; (2) either solicited (*Self Anxiety Reduction Hypothesis, Prediction 2c*) or unsolicited contacts ( *Appeasement Hypothesis* and *Consolation Hypothesis*, *Prediction 3e*); and (3) matched-control condition (MC; absence of aggression). We considered individuals that acted as victim, aggressor or third party in at least three aggressive events. Moreover, we considered the aggressive events in which only one type of triadic affiliation (i.e. solicited or not solicited) with either victim or aggressor occurred. During post-conflict time-window (3-min), we evaluated the numbers of target behaviors before and after the triadic affiliation and then we normalized these numbers over the min of observation before and after the affiliation.

### Statistical analyses

In the case of non-normal distributions of data (Kolmogorov–Smirnov test: *P* < 0.05) we used non-parametric statistics for the analyses (Siegel and Castellan 1988). In particular, we applied the Wilcoxon’s signed rank test corrected for ties for two dependent samples to verify: (i) the occurrence of reconciliation (*Prediction 1a*), TSC (*Prediction 2a*) and TUC (*Prediction 3a*) by comparing the numbers of attracted *vs* dispersed pairs at the individual level; (ii) if kinship could affect the level of reconciliation (*Prediction 1b*), TSCs (*Prediction 2b*) and TUCs (*Prediction 3b*) by comparing the individual proportion of conciliatory/triadic contacts with weakly-related *vs* closely-related companions over the total contacts; (iii) the *Victim Protection Hypothesis* for both solicited and unsolicited triadic contacts (*Predictions 2d* and *3d*) by comparing the hourly frequency of renewed attacks directed to the victim after *vs* no occurrence of either TSC or TUC; (iv) if TUCs could reduce the probability of renewed attacks by the aggressor on other group members (*Appeasement Hypothesis*, *Prediction 3c*), by comparing the number of attacks in presence *vs* absence of TUC.

Via the non-parametric Friedman test for k-dependent samples, we compared the hourly frequencies of aggressor/victim/third-party anxiety-related behaviors across conditions (PC_NOtri_, PC_YESsol_/PC_YESuns_, MC; *Predictions 2c* and *3e*). The Bonferroni-Dunn *post-hoc* test was used for post-hoc pairwise comparisons.

Owing to a normal distribution (Kolmogorov–Smirnov test: *P* ≥ 0.05), we used the parametric paired t-test for two dependent samples to compare the proportion of first post-conflict affiliative contacts initiated by victims *vs* aggressors over the total contacts.

For all the analyses evaluating the occurrence and the possible effects of TSC and TUC, we have considered only those triadic contact events (solicited and unsolicited) that occurred before or in absence of reconciliation.

### Results

### Prediction 1—Reconciliation

#### Prediction 1a

We found that the frequency of attracted pairs was significantly higher than that of dispersed pairs (Wilcoxon test *N*_victims_ = 37, *T* = 0, ties = 21, *p* < 0.001). This result confirms the occurrence of reconciliation in the study group of domestic pigs.

#### Prediction 1b

**Fig. 2:**
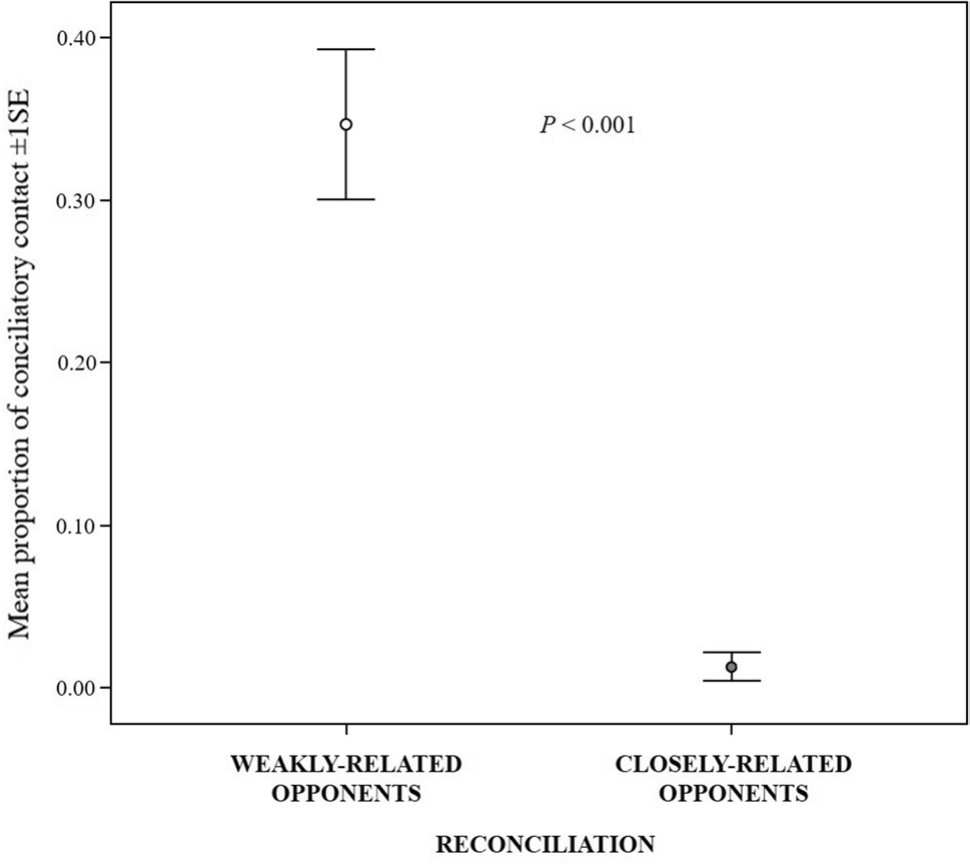
Error bars representing mean proportion of conciliatory contact (± 1SE) between weakly-related and closely-related victim-aggressor dyads

The individual proportion of conciliatory contacts was higher with weakly related than closely related fellows (Wilcoxon test *N*_victims_ = 21, *T* = 1, ties = 1, *p* < 0.001; Fig. 2). Hence, in the study pigs reconciliation did not comply with the *Valuable Relationship Hypothesis* (see Introduction) because it was more frequent between weakly than closely related opponents.

Aggressor and victim started the first affiliative conciliatory contact at comparable levels (Paired t-test *N*_individuals_ = 15, df = 14, *t* = − 1.102, *p* = 0.289; mean affiliations ± SE performed as aggressor 0.14 ± 0.04 and as victim 0.20 ± 0.07).

### Prediction 2—Triadic solicited contacts (TSC)

#### Prediction 2a

When considering the affiliative contacts directed by victims towards third parties, we found that the attracted pairs were significantly higher than dispersed pairs (Wilcoxon test *N*_victims_ = 35, *T* = 34, ties = 11, *p* = 0.001). Hence, this result confirms the occurrence of triadic post-conflict affiliative contacts solicited by the victim (TSC) in the study group. However, 95.2% of affiliative contacts started by victims were not reciprocated by third parties (mean ± SE: not exchanged affiliation 0.95 ± 0.09; exchanged affiliation 0.05 ± 0.03).

When considering affiliative contacts directed by aggressors towards third parties, we found that the attracted pairs did not significantly differ from the dispersed pairs (Wilcoxon test *N*_aggressors_ = 30, *T* = 83.5, ties = 9, *p* = 0.245). Hence, we cannot confirm the occurrence of triadic post-conflict affiliative contacts solicited by the aggressor in the study group. Consequently, *Predictions 2b, 2c* and *2d* could only be tested on the TSC between victim and third party.

#### Prediction 2b

**Fig. 3:**
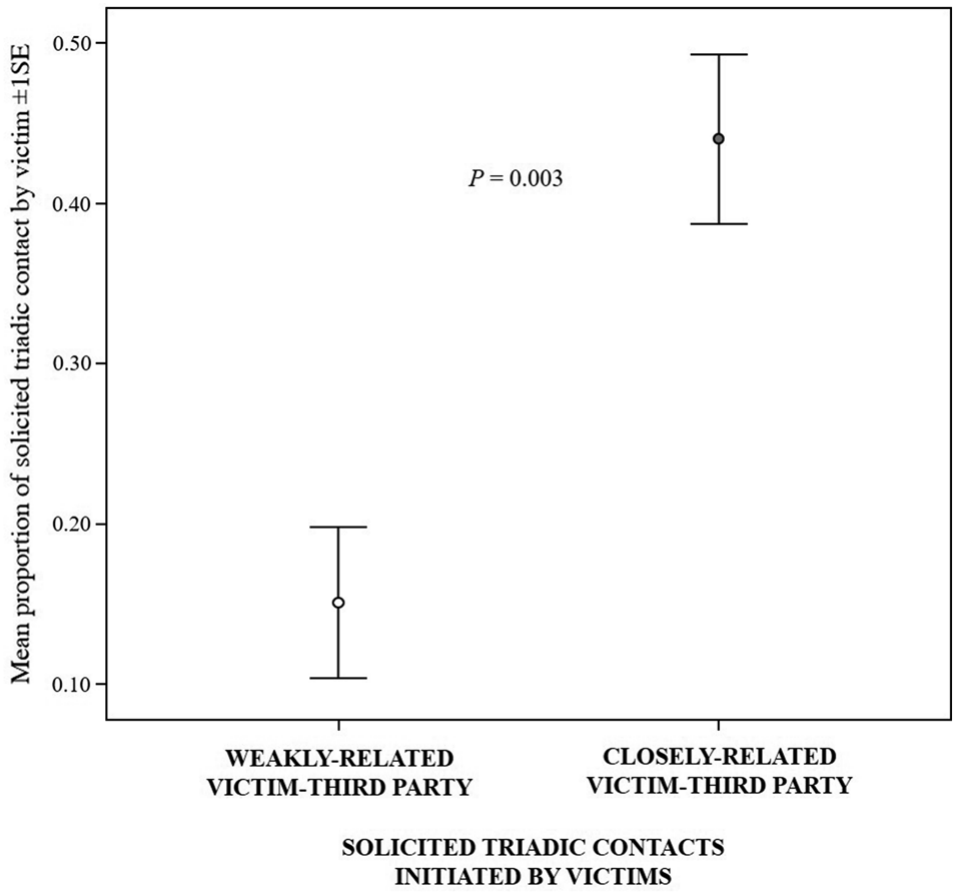
Error bars representing mean proportion of solicited triadic contact (± 1SE) initiated by the victim and directed towards weakly-related and closely-related third party

The proportion of TSCs was higher with closely related than weakly related third parties (Wilcoxon test TSC: *N*_victims_ = 43, *T* = 189, tie = 3, *p* = 0.003; Fig. 3). This finding shows that victims directed their affiliative contacts more frequently towards closely than weakly related third parties.

#### Prediction 2c

**Fig. 4:**
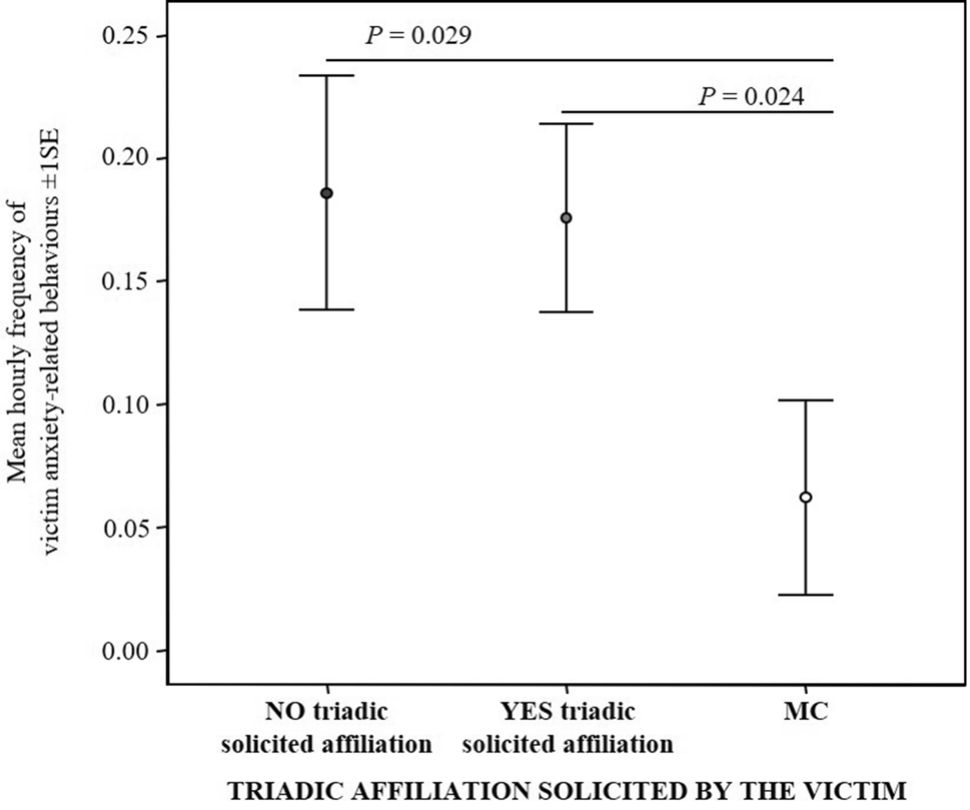
Error bars representing mean hourly frequency (± 1SE) of victim’s anxiety-related behaviors under three conditions: post-conflict period with the absence of solicited contacts initiated by the victim towards a third party (NO triadic solicited affiliation), post-conflict period with the presence of solicited contacts initiated by the victim towards a third party (YES triadic solicited affiliation) and, control period not preceded by any aggression (MC)

The levels of victim’s anxiety-related behaviors significantly differed across the three conditions MC, PC without triadic affiliation and PC with only solicited triadic affiliation (Friedman test *N*_victims_ = 30, *χ*^2^ = 14.026, df = 2, *p* = 0.001; Fig. 4). Particularly, the pairwise comparisons revealed a significant difference between MC-PC without triadic affiliation and MC-PC with only solicited affiliation (Bonferroni-Dunn post-hoc test; MC < PC without affiliation: *Q* = 0.667; *p* = 0.029; MC < PC with affiliation: *Q* = 0.683; *p* = 0.024) but not between PC without triadic affiliation and PC with only solicited affiliation (*Q* = − 0.017; *p* = 1.000). Thus, the levels of victim’s anxiety increased after a conflict but did not significantly decrease after the solicited triadic affiliation.

#### Prediction 2d

The levels of further aggression against the victim by the aggressor were not reduced following TSCs (Wilcoxon test *N*_victims_ = 31, *T* = 19.5, ties = 21, *p* = 0.410). Thus, TSCs did not have a protecting effect towards the victim against further attacks by the aggressor.

### Prediction 3—Triadic unsolicited contacts (TUC)

#### Prediction 3a

When considering the affiliative contacts directed by third parties towards victims (TUC), we found that the attracted pairs were significantly higher than dispersed pairs (Wilcoxon test *N*_victims_ = 41, *T* = 15, ties = 25, *p* = 0.003). When considering the affiliative contacts directed by third parties towards aggressor, we found that the attracted pairs were significantly higher than dispersed pairs (Wilcoxon test *N*_aggressors_ = 31, *T* = 7.5, ties = 14, *p* < 0.001). This finding confirms the occurrence of TUCs directed by third parties towards both victims and aggressors in the study group.

#### Prediction 3b

**Fig. 5:**
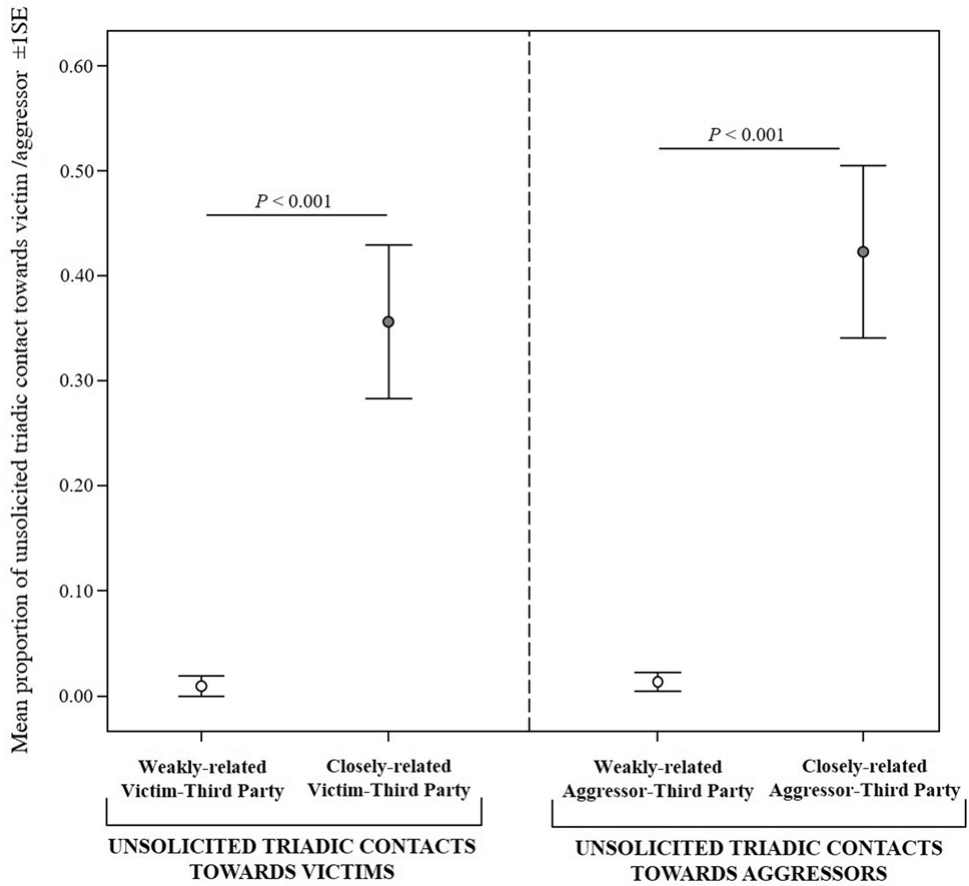
Error bars representing mean proportion of unsolicited triadic contact (± 1SE) initiated by a third party and directed (i) (on the left part of the graph) towards weakly related and closely related victims and (ii) (on the right part of the graph) towards weakly related and closely related aggressors

Close-kin engaged in a higher proportion of TUCs than distant-kin toward either the victim (Wilcoxon test *N*_victims_ = 15, *T* = 2, ties = 0, *p* < 0.001; Fig. 5) or the aggressor (*N*_aggressors_ = 18, *T* = 0, ties = 2, *p* < 0.001; Fig. 5). Hence, third parties offered affiliative contacts more frequently towards a victim or an aggressor that was closely rather than weakly related with them.

#### Predictions 3c

The levels of renewed attacks by the aggressor towards other group members and the victim were lower in presence than in absence of TUCs exchanged with the aggressor (Wilcoxon test *N*_aggressors_ = 13, *T* = 1, ties = 4, *p* = 0.008). Hence, TUCs involving aggressors reduced the risk of renewed aggression, thus possibly having a calming effect.

#### Prediction 3d

The levels of further aggression against the victim by the previous aggressor were comparable in the presence and absence of TUCs exchanged with the victim (Wilcoxon test *N*_victims_ = 13, *T* = 0, ties = 9, *p* = 0.125). This result did not confirm the protecting effect of TUCs directed towards the victim.

#### Prediction 3e

**Fig. 6:**
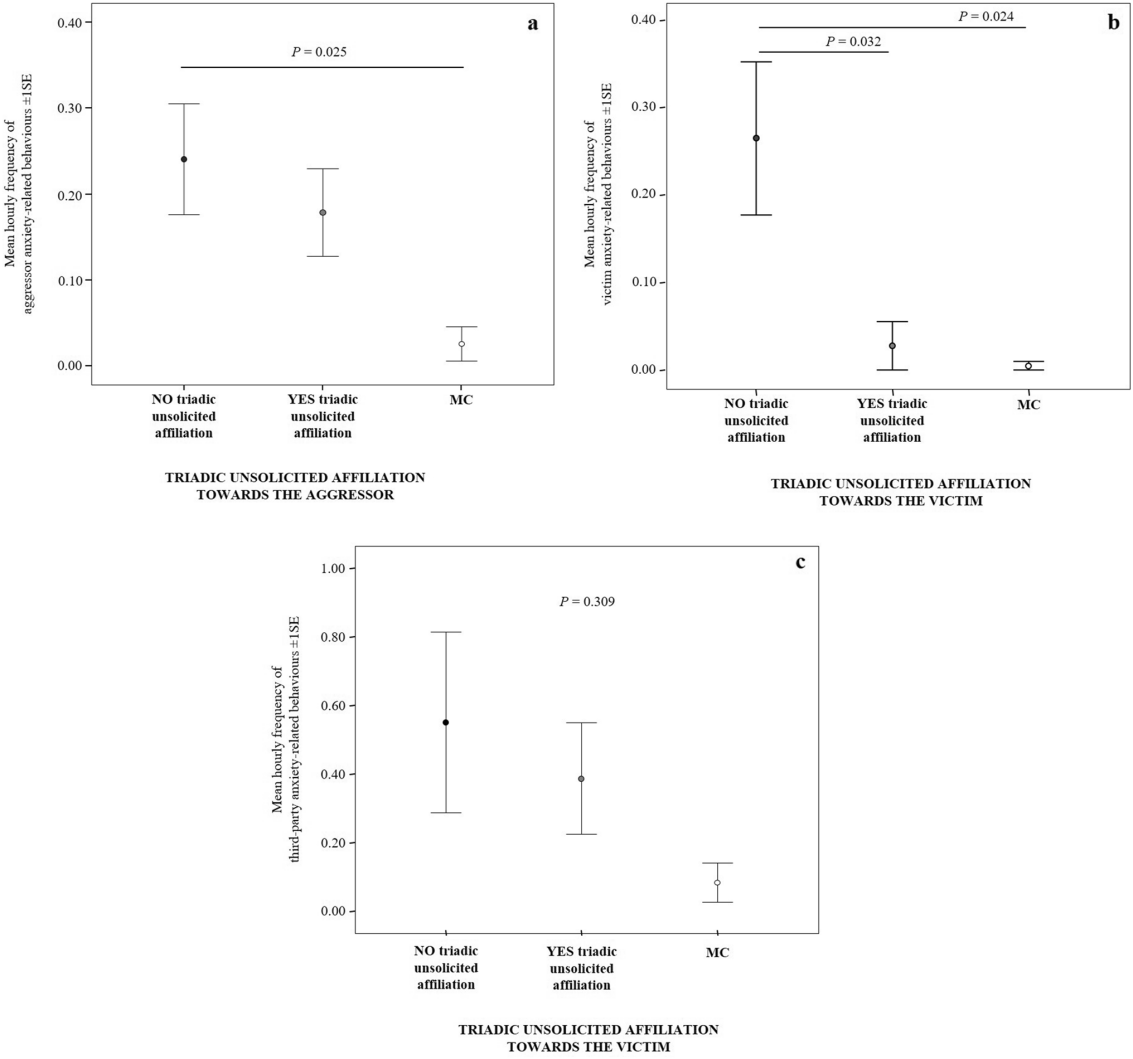
Error bars representing mean hourly frequency (± 1SE) of (**a)** aggressor, (**b)** victim and (**c)** third-party anxiety-related behaviors under three conditions: post-conflict (PC) period with absence (NO) of unsolicited contacts directed to the aggressor (**a**) or to the victim (**b**, **c**), post-conflict period with presence (YES) of unsolicited contacts directed to the aggressor (**a**) or to the victim (**b**, **c**), and control period (MC) not preceded by any aggression

In the case of TUCs towards the aggressor, the levels of aggressor anxiety-related behaviors significantly differed across the three conditions MC, PC without triadic affiliation and PC with only triadic unsolicited affiliation (Friedman test *N*_aggressors_ = 26, *χ*^2^ = 11.841, df = 2, *p* = 0.003; Fig. 6a). There was a significant difference between MC-PC without triadic affiliation (MC < PC without affiliation, Bonferroni-Dunn post-hoc test: *Q* = 0.731; *p* = 0.025) and no difference between MC- PC with only unsolicited affiliation and PC without triadic affiliation- PC with only unsolicited affiliation (MC-PC with affiliation: *Q* = 0.481; *p* = 0.249; PC without affiliation-PC with affiliation: *Q* = 0.250; *p* = 1.000). These findings indicate that the levels of anxiety in the aggressor increased after the conflict but did not significantly decrease after TUCs.

In the case of TUCs towards the victim, the levels of victim anxiety-related behaviors significantly differed across the three conditions (Friedman test *N*_victims_ = 12, *χ*^2^ = 15.500, df = 2, *p* < 0.001; Fig. 6b). The pairwise comparisons revealed a significant difference between MC-PC without triadic affiliation and PC without triadic affiliation-PC with only triadic unsolicited affiliation (Bonferroni-Dunn post-hoc test; MC < PC without affiliation: *Q* = 1.083; *p* = 0.024;  PC without affiliation > PC with affiliation: *Q* = 1.042; *p* = 0.032) but not between MC- PC with only unsolicited affiliation (*Q* = 0.042; *p* = 1.000). As a control, we checked for the variation in the levels of anxiety-related behaviors in the third parties affiliating with victims across the same conditions and we found no significant difference (Friedman test *N*_third-parties_ = 10, *χ*^2^ = 2.348, df = 2, *p* = 0.309; Fig. 6c). Hence, TUCs directed to the victim had a consolatory effect on the victim itself (i.e. a decreased of anxiety-related behaviors). However, TUCs did not reduce the anxiety levels in the third party, thus not showing a self-distress reduction effect.

All results are summarized in Table 1 and the post-conflict strategies found are outlined in Fig. 1.

**Table 1:** Summary of the results obtained in relation to the different post-conflict behaviours showed by domestic pigs, associated with cognitive substrates and tested hypotheses

Post-conflict strategy	Individual mean ± SD number of PC-MC pairs	Involved participants	Social bias	Cognitive substrates	Tested Hypotheses
Reconciliation PC-MC = 149 CCT = 13.6% ± 3.0 SE	4.03 ± 1.61	AG & VC	Yes, preferentially between weakly-related individuals	Second subject participatory capabilities (individual recognition and implicit memory of previously encountered subjects); attributing different social values to others	*Valuable Relationship Hypothesis* rejected
Triadic contacts	4.50 ± 2.05	TSC by AG PC-MC = 134	*	*	*
	4.03 ± 1.60	TSC by VC PC-MC = 141 TCT_sol_ = 20.1% ± 5.0 S.E	Yes, preferentially between closely-related individuals	Intrinsic regulatory mechanisms (change in own experience)	*Self-Anxiety Reduction Hypothesis* rejected
					*Victim Protection Hypothesis* rejected
	3.70 ± 1.00	TUC toward AG by TP PC-MC = 114 TCT_uns_ = 15.9% ± 3.6 S.E	Yes, preferentially between closely-related individuals	Extrinsic regulatory mechanisms (change in others’ experience)	*Appeasement Hypothesis* supported
	3.50 ± 1.30	TUC toward VC by TP PC-MC = 144 TCT_uns_ = 14.3% ± 3.4 S.E		More elements of MNS/PAM based social appraisal	*Victim Protection Hypothesis* rejected
					*Consolation Hypothesis* supported

*AG* Aggressor, *VC* Victim, *TP* Third-Party, *PC-MC* Post-conflict/Matched control pairs, *CCT* Corrected Conciliatory Tendency (mean group value), *TSC* Triadic Solicited Contact, *TUS* Triadic Unsolicited Contact, *TCT* Triadic Contact Tendency (mean group value), *PAM* Perception–Action Model, *MNS* Mirror Neuron System

## Discussion

Our results show that domestic pigs are able to adopt a wide array of post-conflict strategies (as per de Waal 2000). Compared to the Matched Control condition (absence of conflict), in the Post-Conflict condition it was more likely to observe affiliation between former opponents (i.e. reconciliation), between victim and a third party (both solicited—TSC—and unsolicited—TUC—triadic contacts), and between the aggressor and a third party (only TUC) (Table 1; Fig. 1). The enactment of these post-conflict strategies, the different individuals that they involve and the effect that they produce inform the ability to attribute different social values to others, the presence of elements of intrinsic and extrinsic regulation, and possibly social appraisal (*sensu* Zaki and Williams 2013; Walle et al. 2017). These aspects are summarized in Table 1 and detailed below.

### Reconciliation

Reconciliation was present in the domestic pigs under study and was equally started by the aggressor or the victim (*Prediction 1a* supported). Moreover, reconciliation was not randomly distributed across dyads of opponents, even though not in the way it was expected. As a matter of fact, conciliatory contacts were highest between distant-kin rather than close-kin (*Prediction 1b* rejected). Hence, pigs possess the basic individual recognition and implicit memory skills that are necessary to engage in post-conflict reunion and attribute different social values to others. Reconciliation can be affected by the *value* and the *security* of the relationship between subjects (Cords and Aureli 2000). The *value* depends on the benefit that the subjects obtain in terms of affiliation, food-sharing, and social support. *Security* refers to the confidence that a subject has about the solidity of the relation and the predictability of the partner's behavioral response (Cords and Aureli 2000). The damage caused by conflict tends to be lower within highly predictable relationships (Koski 2015). Hence, if relationships are secure, the necessity of reconciling a previous conflict may decrease. In this respect, the relationship between closely related pigs can be highly secure, with little necessity for reconciliation. On the other hand, when a large number of animals live together (as it occurred in our study) weakly associated subjects can gain benefit from limiting their aggressiveness and increasing social tolerance with other in-group competitors for resource monopolization (Andersen et al. 2004; Estevez et al. 2007). In this view, weakly related pigs may strategically use reconciliation to improve the likelihood of tolerant cohabitation.

### Triadic solicited affiliation (TSC)

The pigs under study engaged in TSC, initiated by the victim but not by the aggressor (*Prediction 2a* partially supported). Aggressors—typically in a dominant position—may not gain immediate benefit from affiliating with a third subject after a conflict. The victim started TSCs most frequently with closely related third parties (*Prediction 2b* supported). This result suggests that domestic pigs may possess intrinsic regulation mechanisms, whose success also depends on their relationship with the contacted individual (Zaki and Williams 2013). In this respect, individuals (i.e. former opponents) would look for social contact to possibly regulate their own experience. Our result is consistent with other studies showing that TSCs can be most frequently exchanged between closely associated subjects (Aureli and Schaffner 2002; Fraser et al. 2008; McFarland and Majolo 2012). Because aggression can spread within social groups (social facilitation effect; Wilson 2000; Cordoni and Palagi 2008; Romero et al. 2009), it is probable that victims face less risks of receiving an aggressive reaction by approaching a third parties with which they share a valuable relationship. Intriguingly, TSCs had no influence in reducing post-conflict anxiety in the victim (*Self Anxiety Reduction Hypothesis; Prediction 2c* rejected) and in decreasing the probability of receiving further attacks by the previous aggressor (*Victim Protection Hypothesis*; *Prediction 2d* rejected). From the cognitive point of view, we can hypothesize that TSCs do not require that third parties perceive and actively respond to the distress of others (former opponents) after activating shared representations, as foreseen when third parties show agency and start prosocial behaviors (Decety et al. 2016). In our study, 95.2% of post-conflict affiliative contacts performed by victims were not unreciprocated by third parties. Hence, solicited affiliative contacts were passively accepted by the third party but victims did not actually receive any affiliation from the third party, which sets a marked difference from unsolicited contacts. This situation may explain why TSCs did not lead to successful social regulation. Importantly, this observation opens the question of whether unilateral, unreciprocated contacts can be considered as actual TSCs. This issue has not been tackled by previous literature—which generically refers to the occurrence of affiliative contacts—and should be addressed by future studies.

### Triadic unsolicited affiliation (TUC)

The pigs under study showed TUC offered by third parties to either the victim or the aggressor (*Prediction 3a* supported). Hence, based on the cognitive hypotheses, third parties might able to adopt a non-egocentric perspective and—by shifting from the dyadic to the triadic level—individuals might be able to implicitly share the experiences of others and be attuned to their internal states (Ferrari and Gallese 2007; Rochat et al. 2009; Aaltola 2013; Seyfarth and Cheney 2015). As observed in other social species (Koski and Sterck 2007; Palagi et al. 2008; Palagi and Cordoni 2009; Fraser and Bugnyar 2010; Romero et al. 2010, 2011), TUCs in pigs were more frequently offered to victim or aggressor by close-kin (*Prediction 3b* supported, Table 1). This suggests that a mechanism of social regulation be in place, as the effectiveness of such regulation depends on the interacting subjects (Zaki and Williams 2013). Indeed, depending on the contacted individual (aggressor or victim), TUCs led to different social regulation effects. When TUCs were directed toward the aggressor, they possibly regulated third party’s own experience (intrinsic regulation), as TUCs were followed by a reduction in the levels of renewed attacks toward group members (as per the *Appeasement Hypothesis*; *Prediction 3c* supported). No such reduction was observed when TUCs were offered to the victim (contrary to the *Victim Protection Hypothesis*; *Prediction 3d* rejected). On the other hand, when TUCs were offered to the victim, they were followed by an anxiety level decrease in the victim, which suggests possible extrinsic regulation (*Consolation Hypothesis* Fujisawa et al. 2006; Fraser et al. 2008) *Prediction 3e* partially supported. No anxiety decrease was observed in the aggressors when TUCs were offered to them.

Previous studies on non-human primates have shown that the reduction of the aggressor’s aggressiveness does not necessarily imply the reduction of aggressor’s anxiety (Romero et al. 2009, 2011). Our result suggests that in pigs via TUCs towards aggressors third parties can protect themselves from potential retaliation with a possible benefit for their direct fitness (Cords and Aureli 2000; Schino and Marini 2012).

TUCs toward the victim were followed by a reduction of the victim’s anxiety, which suggests the possible presence of elements of social appraisal in pigs. Indeed, it has been hypothesized that social appraisal allows individuals to assess the valence of a social interaction (e.g. aggression) in which they are not directly involved by detecting the emotional reaction (i.e. distress) of individuals involved in such interaction (Cordoni and Palagi 2008; de Waal and Preston 2017; Walle et al. 2017; Pérez-Manrique and Gomila 2018). As a consequence, the third party may restore the victim emotional homeostasis via affiliation. TUCs in pigs may have a consolatory function, which by definition comes into play when the triadic contacts work in reducing the victim’s arousal (Fraser et al. 2008; Fraser and Bugnyar 2010; de Waal and Preston 2017). As hypothesized by the neurocognitive model of emotional contagion in humans, the third party behavior may lead to the reduction of the discrepancy between the third party (agent) and the other’s internal state (Prochazkova and Kret 2017). An additional element is that anxiety-related behaviors decreased in the victims but not in the third parties affiliating with the victims, which suggests that TUCs might be other- more than self-oriented. However, triadic contacts were mostly directed to close-kin. Thus, they cannot be considered as properly altruistic (sensu Silk and House 2016) as they can lead to indirect kinship benefits.

In sum, this study showed that pigs possess different post-conflict strategies (*sensu *de Waal and van Roosmaleen 1979), including reconciliation and unsolicited triadic contacts. Because the affiliative contacts offered by the victim to a third party remained largely unreciprocated, the actual presence of solicited triadic contact is questionable and future studies should take the reciprocation issue into account. The observation that all the post-conflict strategies were skewed by kinship (and not randomly distributed across dyads) and that the function of unsolicited third-party contacts (renewed aggression probability or anxiety reduction) depended on the contacted individual, point toward complex cognitive abilities, spanning intrinsic/extrinsic socio-emotional regulation and elements of social appraisal. Future study replications—aimed at increasing the generalization of the findings to the species level—should take into account the following main *Constraints on Generality* (*sensu* Simons et al. 2017): (i) only adults were part of the target population, (ii) only the domestic form of *Sus scrofa* (and not the wild counterpart) could be investigated, (ii) group composition slightly changed over time (due to moderate culling) and (ii) males were castrated, which can decrease aggression frequency and/or intensity, and the actual animal sample available for split post-conflict analyses. Despite these limitations, this study has an overall good generalization potential because—due to the extremely low culling rates and high farming standards—data collection could last for over six months on a large mixed breed/sex group of animals ranging semi-freely in a natural habitat (that nonetheless ensured excellent observation conditions), with different kinship degree and an unexpectedly large age span (from early sexual maturity to almost two years of life). Although rarely found in pig farming (even when extensive), when available these conditions are essential to undertake further steps into the investigation of pig post-conflict phenomena and the understanding of their cognitive implications at the species level.

## Supplementary Information

Below is the link to the electronic supplementary material.

Supplementary file1 (XLSX 12 KB)

Supplementary file2 (XLSX 12 KB)

Supplementary file3 (XLSX 12 KB)

Supplementary file4 (XLSX 14 KB)

Supplementary file5 (MP4 161686 KB)

Supplementary file6 (MP4 83357 KB)

Supplementary file7 (MP4 103785 KB)

Supplementary file8 (DOCX 22 KB)

Supplementary file9 (DOCX 15 KB)
