# Supplementary material for: Domestic pigs (Sus scrofa) engage in non-random post-conflict affiliation with third parties: cognitive and functional implications
Source: Anim Cogn. 2022 Nov 8;26(2):687–701. doi: 10.1007/s10071-022-01688-4 (PMC9950185; doi:10.1007/s10071-022-01688-4)
Supplement: Supplementary file 8 — Supplementary file8 (DOCX 22 KB) [file 10071_2022_1688_MOESM8_ESM.docx]

**SUPPLEMENTARY MATERIAL**

**Video_1_solicited_contact_victim**

An example of triadic solicited contact directed by the victim towards a third-party.

Immediately after the conflict, the pig marked with two light blue circles on its back (the victim) flees and shakes her head. Subsequently, the victim moves towards a lactating female (the third-party) that she is foraging and begins a nose-to-nose contact with her.

**Video_2_unsolicited_contact_victim**

An example of triadic unsolicited contact directed towards the victim by a third-party.

Immediately after the conflict, the pig (the victim) flees away and shakes his head while the aggressor moves out of video. An uninvolved pig (the third party) approaches the victim and starts a nose-to-body contact with him.

**Video_3_unsolicited_contact_aggressor**

An example of triadic unsolicited contact directed towards the aggressor by a third-party.

Immediately after the conflict, the pig with blue marks on the back (the victim) flees away. An uninvolved pig (the third party with a light-yellow mark on the back) quickly moves towards the aggressor and begins a nose-to-body contact with him.
