# Supplementary material for: Domestic pigs (Sus scrofa) engage in non-random post-conflict affiliation with third parties: cognitive and functional implications
Source: Anim Cogn. 2022 Nov 8;26(2):687–701. doi: 10.1007/s10071-022-01688-4 (PMC9950185; doi:10.1007/s10071-022-01688-4)
Supplement: Supplementary file 9 — Supplementary file9 (DOCX 15 KB) [file 10071_2022_1688_MOESM9_ESM.docx]

**Table S1 -** Aggressive, affinitive and anxiety-related behaviours of domestic pig (*Sus scrofa*) considered in this study

| **Behavioural pattern** | **Description** |
| --- | --- |
| **AFFINITIVE BEHAVIOURS** | |
| **Rest in contact** | Two pigs sit or lay in contact with one another |
| **Social touching** | A pig touches a fellow with a paw or other body parts, except nose/head |
| **Nose-nose contact** | A pig touches with its nose the nose of a fellow |
| **Nose-body contact** | A pig touches/pushes with its nose a body part of a fellow (excluding nose) |
| **Head-over** | A pig puts its head above the back of a fellow; rest in contact or body contact can after occur |
| **Social rubbing** | A pig rubs over the body of another |
| **AGGRESSIVE BEHAVIOURS** | |
| **Aggressive lifting** | A pig attempts to displace a fellow by lifting or levering it with snout or head |
| **Aggressive biting** | A pig opens its mouth and close its teeth tight on a fellow’s small piece of flesh, including tail |
| **Aggressive mounting** | A pig forces a fellow to move away by rising upon its rear |
| **Aggressive kicking** | A pig projects of one or both hind limbs towards a fellow, striking it |
| **Aggressive pushing** | A pig presses its head, neck, shoulder or body against a fellow thus causing its moving |
| **Aggressive chasing** | A pig pursues a fellow, which flees |
| **Aggressive head-knocking** | A pig lurches or jerks its head hitting a fellow |
| **Fighting** | Two pigs mutually push one another in a head to head orientation. The pattern can involve body-to-body rotation and/or aggressive mounting, lifting, biting, attempt biting, kicking, chasing, pushing, head knocking, high pitched vocalisation, with no interruption lasting more than 10s |
| **ANXIETY-RELATED BEHAVIOURS** | |
| **Body scratching/rubbing** | A pig uses its legs or a substrate to rub part of its body |
| **Vacuum*-*chewing** | A pig chews with empty mouth |
| **Head/Body shaking** | A pig vigorously shakes its head and/or body |
| **Yawning** | A pig performs deep, long inhalation with open mouth |
